# The Evaluation of the Diagnostic Value of a PCR Assay When Compared to a Serologic Micro-Agglutination Test for Canine Leptospirosis

**DOI:** 10.3389/fvets.2022.815103

**Published:** 2022-04-26

**Authors:** Elizabeth A. Martin, Johanna C. Heseltine, Kate E. Creevy

**Affiliations:** ^1^Department of Cardiology, Veterinary Medicine Faculty Intern, Virginia-Maryland College of Veterinary Medicine, Blacksburg, VA, United States; ^2^Department of Small Animal Clinical Sciences, Texas A&M University College of Veterinary Medicine & Biomedical Sciences, College Station, TX, United States

**Keywords:** negative predictive value, positive predictive value, retrospective, sensitivity, specificity

## Abstract

**Objective:**

To evaluate PCR assay sensitivity and specificity compared to that of microscopic agglutination test (MAT) for diagnosis of canine leptospirosis. Electronic records search was performed to identify dogs with results for both PCR and MAT testing for leptospirosis.

**Methods:**

All dogs were clinically ill. Diagnosis of leptospirosis was defined as an unvaccinated dog with a positive MAT titer of ≥1:800 or a vaccinated dog or dog with an unknown vaccination status with a positive MAT titer of ≥1:1,600. Diagnosis of leptospirosis was excluded based on MAT titer <1:800 on both the initial and convalescent samples or an initial MAT titer <1:800 and an alternative definitive diagnosis.

**Results:**

Forty-nine samples (urine, *n* = 39; blood, *n* = 10) were evaluated. Leptospirosis was diagnosed in 17 dogs and excluded in 26 dogs. Urine PCR assay demonstrated sensitivity of 69.2%, specificity of 100%, positive predictive value of 100%, and negative predictive value of 86.6%. Blood PCR assay demonstrated sensitivity of 25%, specificity of 100%, positive predictive value of 100%, and negative predictive value of 25%. Overall PCR sensitivity was 52.4%, specificity was 100%, positive predictive value was 100%, and negative predictive value was 73.7%.

**Conclusions:**

PCR assay performed on urine or blood has high specificity and positive predictive value when compared to MAT for diagnosis of clinical canine leptospirosis. Sensitivity and negative predictive value are moderate to low, so PCR testing should be performed in conjunction with paired MAT testing for canine leptospirosis. Prior antibiotic therapy does not preclude the use of the PCR test.

## Introduction

Leptospirosis is an emerging zoonotic disease with a worldwide distribution. Multiple serovars within pathogenic species *Leptospira interrogans* and *Leptospira kirschneri* can result in clinical disease in dogs (1). Dogs serve as accidental hosts when exposed to bacterial organisms in urine from infected animals, or contaminated water or soil. Leptospires enter the body most commonly through intact mucous membranes or abraded skin. Other forms of transmission include bite wounds, venereal contact, and ingestion of contaminated tissues, soil, or fomites (1). Serovars that most commonly infect dogs and lead to clinical disease in the United States include Autumnalis, Bratislava, Canicola, Grippotyphosa, Icterohaemorrhagiae, and Pomona (1, 2).

In dogs, infection with *Leptospira* can result in a wide range of clinical signs depending on severity of infection and infecting strain. Infection can be subclinical, result in mild signs of disease, or result in serious illness with severe injury of one or more organs and sometimes death (1). Clinical presentations include renal injury, hepatic injury, and uveitis. Pulmonary hemorrhage or abortion are rare presentations in the United States. Fever can be present early in the disease process. Dogs presenting with renal or hepatic failure often exhibit signs such as vomiting, diarrhea, inappetence, abdominal pain, icterus, polyuria, and polydipsia (1).

Techniques used in the diagnosis of leptospirosis include serology, PCR, darkfield microscopy, histopathology, and bacterial culture (1). Clinically, serology using the microscopic agglutination test (MAT) and PCR assays are used predominantly. Leptospiremia develops during the first phase of infection, which lasts 3–10 days. The bacteria then migrate to the kidneys and are shed in the urine during the second phase of infection, which ranges from 7–14 days. This shift can affect the sensitivity of PCR testing based on the phase of infection and the sample tested (1).

In recent decades, MAT has been the gold standard for the diagnosis of canine leptospirosis; however, it has several disadvantages. The test is performed by incubating live cultures of various leptospiral serovars with serial dilutions of patient serum and assessing for agglutination by darkfield microscopy. The highest serum dilution causing 50% agglutination is the value reported. The MAT is complicated to perform requiring live cultures of zoonotic, pathogenic serovars and considerable expertise for interpretation (1). Sensitivity of MAT at the time of a dog's presentation has been reported to be 22–67% (3). Commonly, in an unvaccinated dog, a single titer ≥1:800 for any serovar is considered positive. In a vaccinated dog, a single titer of ≥1:1600 for any serovar is considered positive (2). The sensitivity of MAT increases when it is used as a paired test, with convalescent titers performed 2–4 weeks after the initial titer. A 4-fold change in antibody titer is indicative of recent infection (1). Generally, titers will change more slowly or will have no change following vaccination or chronic infection (1). Post-vaccination titers are commonly low (≤1:400) and persist for approximately 3 months or less. However, one study showed that post-vaccination titers can increase to ≥1:800 and even to ≥1:1,600 for serogroup Autumnalis for at least 4 weeks (4). The fact that MAT results are individually reported for each tested serovar was previously considered an advantage of MAT; however, cross-reactivity among serovars is common (2). For these reasons, and because infecting serovar is not directly relevant to diagnosis or therapy, MAT is no longer utilized to recognize the infecting serogroup (5). PCR assays detect leptospiral nucleic acid in urine, blood, or certain other body fluids or tissue samples (6). PCR testing for canine leptospirosis is gaining recognition as it may have the advantage of identifying infection earlier in the course of disease compared to serologic testing. In dogs, PCR results have been shown not to be influenced by recent vaccination with two separate leptospirosis four-way vaccinations (7). Based on the progression of leptospiremia to leptospiruria, the recommendation for use of PCR is to select the specimen for testing based on the phase of the disease process; however, this can be difficult to determine in a clinical patient. When onset of infection is unknown, the recommendation is to submit both urine and blood samples (7). A limited number of studies have evaluated PCR testing for canine leptospirosis. In an American study, eight dogs were diagnosed with leptospirosis and all had positive urine PCR results (8). In one study, blood and/or urine PCR results from dogs with leptospirosis-associated and non-leptospirosis acute kidney injury (AKI) were negative for all samples, and this was attributed to the prior administration of antimicrobials (9). In another study, PCR was positive in 41/500 (8.2%) dogs presenting serially to a veterinary teaching hospital, while only four of the dogs had clinical illness attributable to leptospirosis, which may suggest shedding in the absence of clinical signs or may suggest false positive results obtained by urine PCR (6). In a Brazilian study, eight dogs were diagnosed with leptospirosis via MAT, and four of those had a positive PCR result on urine and/or blood. Ten dogs had positive PCR results with a negative MAT titer (10). Given these results, it has been suggested that both the MAT and PCR assay should be performed when attempting to diagnose canine leptospirosis (6, 10).

The purpose of this study was to investigate the specificity and sensitivity of PCR testing on blood and/or urine compared to MAT testing for the diagnosis of naturally occurring canine leptospirosis.

## Methods

### Case Selection

A medical record search at the Texas A&M University Veterinary Teaching Hospital (TAMU VTH) was used to identify dogs with both MAT and PCR results for leptospires between 2008 and 2019. Each record was assessed to determine whether leptospirosis had been definitively diagnosed, definitively excluded, or remained uncertain, as follows. Leptospirosis was diagnosed based on a single titer >1:800 for any serovar in unvaccinated dogs, or >1:1600 for any serovar in vaccinated dogs and dogs with unknown vaccine status, or a 4-fold change in titer for any serovar between the acute and convalescent titers. Leptospirosis was excluded when neither initial nor convalescent titers were positive using the same criteria and there was not a 4-fold increase between the two titers. Additionally, leptospirosis was ruled out when a single MAT titer was negative, and an alternative diagnosis was made that was considered by the attending clinician to be the cause of the patient's illness. Leptospirosis remained uncertain in cases not meeting the above criteria, and those records were excluded from further analysis. MAT titers were measured for the following serovars: Pomona, Icterohaemorrhagiae, Canicola, Grippotyphosa, Hardjo, Bratislava, Autumnalis, and Serjoe. MAT was performed at the Texas A&M Veterinary Medical Diagnostic Laboratory (College Station, TX). Over the time period of this study, more than one commercial laboratory performed the PCR test^1^; samples were sent to the commercial laboratory being used by the TAMU VTH at the time each patient was seen, and sample interpretation was based on that laboratory's report.

Additional information collected from each record included signalment, clinical signs, duration of clinical signs, leptospirosis vaccination status, date PCR and MAT were performed, specimen(s) used for PCR testing, dates of admission and discharge, body temperature at presentation, and whether the patient had received antibiotics before sample collection.

### Data Analysis

Diagnostic accuracy was defined by sensitivity, specificity, positive predictive value (PPV), and negative predictive value (NPV) for each sample. Based on these obtained values, point prevalence was calculated. The diagnostic accuracy was determined using the following formulas:


$$\begin{array}{l} \text{Sensitivity }(\%)\text{ = True positives/ }(\text{true positives}\\ \;+\text{ false negatives})\;\times \text{ 100}\%\\ \text{Specificity }(\%)\text{ = True negatives/ }(\text{false positives}\\ \;+\text{ true negatives})\;\times \text{ 100}\%\;\\ \text{Positive predictive value }(\%)\text{ = True positives/ }(\text{true}\\ \;positives\;+\text{ false positives})\;\times \text{ 100}\%\;\\ \text{Negative predictive value }(\%)\text{ = True negatives/ }(\text{false}\\ \;negatives+\text{ true negatives})\;\times \text{ 100}\%\;\\ \text{Prevalence }(\%)\text{ = }(\text{True positives + false negatives})\;\\ \text{ /}(\text{true positives + false negatives + false positives}\\ \;+\text{ true negatives})\;\times \text{ 100}\%\; \end{array}$$

## Results

During the eleven-year time period, 90 dogs were identified who had both leptospiral MAT and PCR testing performed. Of these, 45 were excluded due to lack of definitive diagnosis or exclusion of leptospirosis according to study parameters and two were excluded because diagnosis was made at necropsy with tissue sampling collected post-mortem. Forty-three dogs were identified that met the inclusion criteria. There were five Labrador retrievers, five Chihuahuas, four boxers, three mixed breeds, two Yorkshire terriers, two Dachshunds, two American pit bull terriers, and one each of miniature Australian shepherd, Siberian Husky, Newfoundland, Catahoula hog dog, miniature Dachshund, Queensland heeler, Walker hound, Maltese, Wheaten terrier, West Highland terrier, great Dane, wirehaired pointing Griffon, black-mouth cur, miniature schnauzer, German short-haired pointer, Rottweiler, shih tzu, Shetland sheepdog, Australian heeler, and Llewellin setter. There were 23 female dogs with 22 of those being spayed. There were 20 male dogs with 14 of those being castrated. The average age was 6.3 years with a median of 6 years (range 0.5–15 years). Leptospirosis vaccination status of approximately half of the dogs was unknown (21/43 48.8%). There were more unvaccinated dogs (15/43; 34.9%) than vaccinated dogs (7/43; 16.3%). All dogs were clinically ill. The average duration of clinical signs was 7 days with a median of 5 days (range 1 −28 days). The most commonly reported clinical signs were vomiting (30/43; 69.8%), lethargy (19/43; 44.2%), anorexia (14/43; 32.6%), and hyporexia (12/43; 27.9%). Less common clinical signs included polyuria with polydipsia (5/43; 11.6%), and diarrhea (5/43; 11.6%) with one dog (2.3%) presenting with abnormal mentation. There were 10 dogs (23.3%) that had elevated liver enzymes and 34 dogs (79.1%) with azotemia on the day of presentation.

Of the 43 dogs enrolled, 49 samples were PCR tested, including ten blood samples and 39 urine samples. There were six dogs that had both urine and blood samples submitted for PCR. Table 1 shows the PCR results for urine samples, blood samples, and combined urine and blood. Sixteen dogs were definitively diagnosed with leptospirosis based on single MAT and one was definitively diagnosed based on paired titers. Twenty-six dogs had leptospirosis definitively excluded; 23 dogs had a negative single MAT with an alternate diagnosis and three dogs had negative paired titers. For the six dogs with both a urine and blood PCR sample submitted, two dogs had a positive urine PCR, negative blood PCR, with positive MAT. One dog had a positive urine PCR, negative blood PCR, positive MAT, and positive convalescent titer. Two dogs had a negative urine PCR, negative blood PCR, and a negative MAT. One dog had a negative urine PCR, negative blood PCR, and positive MAT result.

**Table 1:** PCR assay results compared with microagglutination titer (MAT) results in total, from blood alone, and from urine alone in dogs of this report.

**Total samples**		**Leptospirosis**	**Other disease**
		MAT positive	MAT negative
	PCR +	11	0
	PCR -	10	28
**Blood samples**		**Leptospirosis**	**Other disease**
		MAT positive	MAT negative
	PCR +	2	0
	PCR -	6	2
**Urine samples**		**Leptospirosis**	**Other disease**
		MAT positive	MAT negative
	PCR +	9	0
	PCR -	4	26

Confirmed alternate diagnoses included grape toxicity, snake bite, copper-associated chronic hepatitis with secondary Fanconi syndrome, chronic pericholangiohepatitis and prostatic hyperplasia, systemic granulomatous vasculitis, Addison's disease, *Escherichia coli* urinary tract infection, *Escherichia coli* pyelonephritis with oliguric renal failure, severe chronic membranoproliferative glomerulopathy, large cell lymphoma, renal amyloidosis, distal renal tubular acidosis of unknown etiology with protein-losing nephropathy, stage four chronic kidney disease, stage three congenital renal dysplasia, unstaged congenital renal dysplasia, *Escherichia coli* cholangitis, right renal agenesis with left renal ectopic ureter and chronic interstitial nephritis, hepatic lymphoma, hepatic lymphoma with chronic interstitial nephritis and glomerulosclerosis, protein-losing nephropathy with oliguric renal failure secondary to severe amyloidosis, obstructive intraluminal urinary bladder mass with secondary urinary tract infection, acute gastroenteritis with acute severe hepatopathy, and schistosomiasis.

Table 2 shows the sensitivity, specificity, PPV, and NPV for the PCR test. When analyzing solely urine samples, sensitivity was 69% and specificity was 100%. The PPV was 100% with NPV of 87%. When analyzing solely blood samples, sensitivity was 25% and specificity was 100%. The PPV was 100% with NPV of 25%. When urine and blood samples were analyzed together, sensitivity was 52% and specificity was 100%. The PPV was 100% and the NPV was 74%. The overall point prevalence calculated based on the total number of samples was 43%. It is possible to calculate the in-parallel sensitivity of performing an acute MAT and a PCR on blood/urine concurrently. Using the previously reported sensitivity for MAT of 22–67% (3), and assuming a specificity of 100% for the gold standard, with the PCR sensitivity and specificity derived from our study, 52 and 100% respectively, the in-parallel sensitivity for both tests is 62–84%.

**Table 2:** Sensitivity, specificity, positive predictive value (PPV), and negative predictive value (NPV) for the PCR assay in total, from blood alone, and from urine alone in dogs of this report.

**Total**			
Sensitivity:	0.52	PPV:	1
Specificity:	1	NPV:	0.74
**Blood**			
Sensitivity:	0.25	PPV:	1
Specificity:	1	NPV:	0.25
**Urine**			
Sensitivity:	0.69	PPV:	1
Specificity:	1	NPV:	0.87

Antibiotics were administered to 22 dogs prior to sample collection for leptospirosis diagnostics. The most common antibiotics administered individually were ampicillin/amoxicillin (nine dogs), doxycycline (four dogs), and ampicillin-sulbactam/amoxicillin-clavulanate (two dogs). Metronidazole, fluoroquinolones, and trimethoprim sulfamethoxazole were given to one dog each. Four dogs received a combination of antibiotics prior to testing. One dog received amoxicillin-clavulanate and ampicillin, one dog received enrofloxacin and amoxicillin-clavulanate, one dog received ampicillin and doxycycline and one dog received enrofloxacin and ampicillin. From these 22 dogs, there were 24 samples submitted for PCR assay; 21 urine samples, and three blood samples. The PCR results from the 22 dogs given antibiotics prior to sample collection included 14 true negative (all urine samples), six true positives (five urine samples, one blood sample), and four false negatives (two urine samples, two blood samples).

## Discussion

The ability to implement early antimicrobial and supportive care for the treatment of canine leptospirosis depends upon a timely and reliable diagnosis. MAT has been the gold standard in the diagnosis of leptospirosis in dogs for many years. As MAT relies on the presence of antibodies in blood, results are commonly negative early in the infection, prior to antibody production (1). Because of this, there has been increasing interest in the use of leptospirosis PCR testing, which may provide a diagnosis earlier in the course of disease. In our study, leptospirosis was diagnosed based on the presence of clinical signs that prompted the attending clinician to test for leptospirosis, and positive MAT results. A cut off of ≥1:800 for unvaccinated dogs and ≥1:1600 for vaccinated dogs/dogs with unknown vaccine status was selected based on previous reports (4, 9). In general, post-vaccination titers are low ( ≤ 1:400) and persist for approximately 3 months or less. However, in one study, post-vaccine titers for the serogroup Autumnalis increased as high as ≥1:1,600 for at least 4 weeks (4). None of the dogs included in our leptospirosis group was diagnosed based on a positive titer to the serogroup Autumnalis alone. In the study reported here, the sensitivity of the PCR assay was greatest when performed on urine (69.2%) while the overall sensitivity of PCR testing using either blood or urine was 52.4%. The sensitivity of PCR reported here is lower than some previous reports. A study in 2003 evaluated the sensitivity and specificity of PCR on urine samples of 132 dogs with clinical signs supportive of leptospirosis, including eight dogs that were diagnosed with leptospirosis, and reported a sensitivity of 100%, a specificity of 88.3%, a PPV of 33%, and a NPV of 100% with the overall prevalence calculated to be 5.5% during the study period (8). In 2018, a study of 33 dogs with clinical signs compatible with leptospirosis reported that of eight dogs diagnosed with leptospirosis based on clinical signs and an MAT titer >1:800, four (50%) were PCR positive on blood and/or urine samples (10).

Antibiotic therapy may impact PCR results. In a 2013 study of MAT and PCR results among dogs referred to a veterinary teaching hospital for AKI, 20 dogs were diagnosed with acute leptospirosis and only one had a positive urine PCR result; in fact, sample collection for the PCR assay was discontinued during the study due to low diagnostic yield (9). Every dog in that study had received antimicrobials from its primary care veterinarian and had been treated by its primary care veterinarian for a median of 4 days prior to referral; the lack of detection of leptospires on urine PCR was attributed to this prior antimicrobial administration. Interestingly, in our study, among dogs with a definitive diagnosis of leptospirosis that had received prior antimicrobial therapy, there were six true positives (five urine samples, one blood sample) and four false negatives (two urine samples, two blood samples), indicating that PCR results may be positive despite prior antimicrobial therapy. PCR has the potential to be more sensitive than MAT in the acute phase of infection because it detects pathogen nucleic acid rather than antibodies. In a study in which urine leptospiral PCR testing was performed in 500 dogs presenting serially, regardless of health status, four dogs had a diagnosis of clinical leptospirosis and three of these four (75%) had a positive PCR result (6). However, an additional 32 dogs had positive urine PCR results in the absence of positive titers on MAT for any serovar. The authors postulated that dogs may shed leptospires in the urine in the absence of either clinical signs or seroconversion. This finding highlights the importance of understanding the pathophysiology of an infection when assessing the role of new diagnostic tests.

In our study, the PCR assay was least sensitive when performed on blood (25%). This may indicate that the dogs were no longer leptospiremic at the time of testing. One limitation of our study is the small number of dogs that had blood submitted for PCR (*n* = 10). For six blood samples there was a concurrent urine sample submitted. In two dogs, both the urine and blood PCR samples were negative with an alternate diagnosis found. One dog had a false negative urine and blood PCR sample compared to concurrent positive MAT. Three dogs had a true positive urine PCR sample and negative PCR blood sample (which could represent a false negative, or the clearance of leptospiremia by this point in each dog's course of illness). As a result, the authors conclude that both blood and urine samples should be submitted for PCR testing, as has been previously recommended (1). Furthermore, based on the in-parallel sensitivity, acute evaluation of both MAT and PCR simultaneously increases the likelihood of making the diagnosis of leptospirosis. This could avoid the delay associated with awaiting results from the convalescent titer to make the diagnosis.

In our study the specificity of *Leptospira* PCR testing on blood and/or urine was 100% and the PPV was 100%. This suggests that PCR can be used to diagnosis leptospirosis in clinically ill dogs. This finding must be applied with care. In our study, only dogs for which leptospirosis had been definitively diagnosed or definitively excluded were evaluated. In the prior study of 132 clinically ill dogs, the specificity of urine PCR was only 88.3% with a PPV of 33%. However, this study analyzed dogs with uncertain leptospirosis status as members of the group without a diagnosis of leptospirosis; it is possible that some of these dogs were misclassified (8). In the prior study of 33 clinically ill dogs, eight (24.4%) of which were confirmed to have leptospirosis based on MAT results, an additional 14 dogs (42.4%) had positive PCR in blood (*n* = 2), urine (*n* = 8) or both (*n* = 4) with negative (<1:800) acute MAT (10). Convalescent titers were not performed in all dogs in that study, and it is possible that some cases of leptospirosis were misclassified on acute MAT titer alone. Taken together, these findings confirm the fact that diagnostic accuracy of any test will vary based on the population tested and the criteria used to establish a definitive diagnosis.

As this study was retrospective in nature, it may be vulnerable to other confounding factors. Decisions about which patients to test for leptospirosis were made by attending clinicians based on combinations of factors that cannot be fully elucidated retrospectively. As the PCR assays were performed at two different commercial laboratories, there is the potential for inter-assay variability that could have affected the overall sensitivity and specificity of the data analyzed. Also, it has been shown that positive PCR results can occur among dogs that are actively shedding the bacteria, in the absence of clinical signs (6). Since all the dogs in this study were clinically ill, it is less likely that PCR identification of leptospires was incidental, but that possibility cannot be excluded. Finally, this study utilized a higher MAT cutoff to establish a diagnosis of leptospirosis (≥1:800 for unvaccinated dogs and ≥1:1600 for vaccinated dogs/dogs with unknown vaccine status) in comparison to some previously conducted studies, which could affect the direct comparison of our results (8, 9). We acknowledge that some studies (11) have reported even higher MAT titers among apparently healthy vaccinated dogs, and therefore the optimal cut-off for MAT in all vaccinated dogs may even be higher than the cut-offs used here. As the exact timing, dosage, compliance, and reasoning for antibiotic drug choice was not available in the medical records, a true evaluation of antimicrobial therapy on PCR results was not obtained.

In conclusion, we recommend that for the clinical diagnosis of canine leptospirosis, PCR testing should be performed on both blood and urine. Results should be evaluated in conjunction with MAT testing, particularly including a convalescent titer when initial diagnostics are not confirmatory. Additionally, our results suggest that PCR testing may have clinical utility even if the patient has received prior antimicrobial therapy.

## Data Availability Statement

The raw data supporting the conclusions of this article will be made available by the authors, without undue reservation.

## Ethics Statement

Ethical review and approval was not required for the animal study because as the research was retrospective, the data was taken from medical records with no direct contact with animals.

## Author Contributions

EM performed the data collection process and analyzed the data with assistance from KC and JH. All authors have read and accepted the manuscript as it is presented to the journal.

## Conflict of Interest

The authors declare that the research was conducted in the absence of any commercial or financial relationships that could be construed as a potential conflict of interest.

## Publisher's Note

All claims expressed in this article are solely those of the authors and do not necessarily represent those of their affiliated organizations, or those of the publisher, the editors and the reviewers. Any product that may be evaluated in this article, or claim that may be made by its manufacturer, is not guaranteed or endorsed by the publisher.

## Footnotes

^1^IDEXX Laboratories Inc, Westbrook, ME; Texas Veterinary Medical Diagnostic Laboratory (TVMDL), College Station TX.
